# Marginal and internal fit and intaglio surface trueness of interim crowns fabricated from tooth preparation of four finish line locations

**DOI:** 10.1038/s41598-021-93455-7

**Published:** 2021-07-06

**Authors:** Keunbada Son, Young-Tak Son, Ji-Min Lee, Kyu-Bok Lee

**Affiliations:** 1 Department of Dental Science, Graduate School, Kyungpook National University, Daegu, Republic of Korea; 2 Advanced Dental Device Development Institute, Kyungpook National University, Daegu, Republic of Korea; 3 Department of Prosthodontics, School of Dentistry, Kyungpook National University, Daegu, Republic of Korea

**Keywords:** Health care, Medical research

## Abstract

This study evaluated the marginal and internal fit and intaglio surface trueness of interim crowns fabricated from tooth preparation scanned at four finish line locations. The right maxillary first molar tooth preparation model was fabricated using a ceramic material and placed in four finish line locations (supragingival, equigingival, subgingival, and subgingival with a cord). Intraoral scanning was performed. Crowns were designed based on the scanned area. Interim crowns were fabricated using a stereolithography three-dimensional (3D) printer (N = 16 per location). Marginal and internal fit were evaluated with a silicone replica technique. Intaglio surface trueness was evaluated using a 3D inspection software. One-way analysis of variance and Tukey HSD test were performed for comparisons (α = 0.05). The marginal and internal fit showed significant differences according to locations (*P* < 0.05); the marginal fit showed the best results in the supragingival finish line (*P* < 0.05). Intaglio surface trueness was significantly different in the marginal region, with the highest value in the subgingival location (*P* < 0.05). Crowns fabricated on the subgingival finish line caused inaccurate marginal fit due to poor fabrication reproducibility of the marginal region. The use of an intraoral scanner should be decided on the clinical situation and needs.

## Introduction

The introduction of chairside dental computer-aided design and computer-aided manufacturing (CAD/CAM) systems in dental clinics is rapidly increasing^1–3^. Therefore, the use of intraoral scanners for impression acquisition is increasing, and many studies have tried to verify scanning accuracy under various clinical conditions^4–6^. To verify the intraoral scanner, the scanning accuracy is also evaluated, but many previous studies have evaluated the marginal and internal fit of dental prosthesis fabricated using an intraoral scanner for application to dental clinics^7–10^. The marginal fit of dental prosthesis considers the clinically acceptable range within 120 µm for reasons such as secondary caries, cement dissolution, and gingival inflammation^11–13^.

In chairside dental CAD/CAM systems, CAM can be divided into milling and additive technologies, and three-dimensional (3D) printing (additive technology) is widely used in the fabrication of interim dental prostheses^14–16^. Previous studies have evaluated 3D trueness to verify the dimensional change of the intaglio surface of the fabricated dental prosthesis^17–19^. Previous studies can be different depending on what is designated as a reference model, such as the manufacturing precision of 3D printers^20^ and the adjustment of the intaglio surface of the crown in the oral cavity^21^.

The intraoral scanner has advantages of superior convenience, fast acquisition time of the virtual model, and superior accuracy (based on scanning for single unit) compared with the conventional method^22–25^. However, the use of an intraoral scanner for fixed dental prosthesis still requires a solution from a clinical perspective^26,27^. Since scan distortion occurs from the starting tooth of the intraoral scanning, the possible scan range for fixed dental prosthesis is still limited^28^. Moreover, factors such as the difference in accuracy according to the type of scanner^29,30^, inaccuracy of the scan due to the patient’s saliva^31^, and effect of ambient light in dental clinics on the accuracy^32^ still require consensus.

In a dental clinical environment, various finish line locations of tooth preparations are often required for fixed dental prosthesis^33^. However, a previous study verified the difference in the scanning accuracy according to the finish line locations of tooth preparation, and inadequate scanning accuracy was reported for clinical application at the subgingival finish line^34^. These results indicate that the finish line locations of tooth preparation may affect dental prostheses fabricated using intraoral scanners; however, these studies are still limited.

Therefore, this study aimed to evaluate the marginal and internal fit and intaglio surface trueness of interim crowns fabricated from tooth preparations of four finish line locations, namely, supragingival, equigingival, subgingival, and subgingival with a cord finish line. The null hypothesis indicates that the marginal and internal fit and intaglio surface trueness of interim crowns fabricated at the four finish line locations did not differ significantly.

## Methods

### Sample preparation

To prepare a reference model of tooth preparation, the right maxillary first molar was milled under the following conditions (occlusal reduction, 1.5 mm; axial reduction, 1.2 mm; finish line design, chamfer) using a milling unit (Ezis HM; DDS, Seoul, Republic of Korea). To reproduce the oral environment, a lithium disilicate ceramic (IPS e.max CAD; Ivoclar Vivadent AG, Schaan, Liechtenstein) having a transparency similar to that of natural teeth was used. After the crystallization process according to the manufacturer’s recommendations, to reduce the gloss of the surface, the surface was polished using diamond rotary instruments (852.FG.010; Jota AG, Rüthi, Switzerland). The adjacent teeth were manufactured using a 3D printer (Megprinter; Megagen, Daegu, Republic of Korea), transparent silicone (Elite Transparent; Zhermack, Badia Polesine, Italy) was used to reproduce the oral environment, and red pigment (406 red; Shinhan, Seoul, Republic of Korea) was used and replace with semitransparent silicone.

### Fabrication of interim crowns and evaluation of intaglio surface trueness

To determine the number of interim crowns (sample size) to be fabricated per finishing line locations, three pilot experiments were performed prior to this study. Based on the results of the pilot experiment, the sample size was determined using power analysis software (G*Power v3.1.9.2; Heinrich-Heine-Universität Düsseldorf, Düsseldorf, Germany) (N = 16; effect size [f] = 0.63; actual power = 99.11%; power = 99%; α = 0.05).

**Figure 1:**
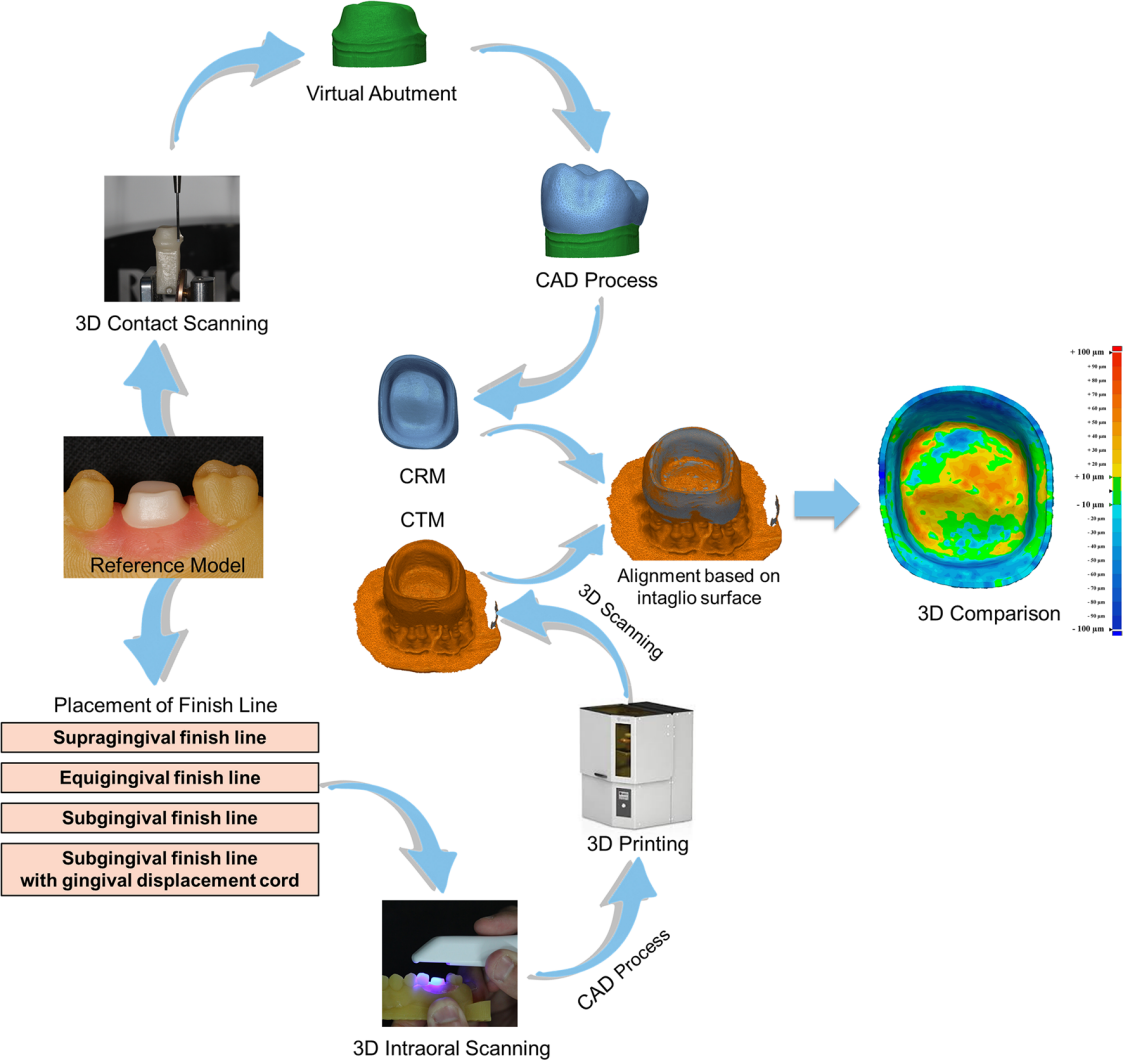
Procedure for intaglio surface trueness of interim crowns fabricated from tooth preparation scanned at four finish lines.

To obtain a reference virtual model of tooth preparation, a precise surface scanning was performed using a contact scanner (DS10; Renishaw plc, Gloucestershire, UK) (Fig. 1). To obtain a high-resolution virtual model, five standard tessellation language (STL) files were acquired through contact scanning and five STL files were merged after optimization alignment by using a 3D mesh software program (Geomagic Design X; 3D Systems, Rock Hill, USA).

The reference model of tooth preparation was adapted to the conditions of each group and fixed to the reference model without movement. The supragingival finishing line was located approximately 0.5 mm above from level of the gingiva, whereas the subgingival finishing line was located approximately 0.5 mm below from the level of the gingiva. The equigingival finishing line was located at the level of the gingiva. Additionally, at the subgingival finishing line, a gingival displacement cord (# 2 Ultrapak; Ultradent, South Jordan, UT, USA) was packed into the gingival sulcus below the finishing line. The depth of the subgingival finishing line was confirmed using a periodontal probe (CP 15 UNC; HU-Friedy, CHI, USA).

To obtain a test virtual model of tooth preparation, an intraoral scanner (i500; MEDIT, Seoul, Republic of Korea) was used to scan a reference model at the supragingival, equigingival, subgingival, and subgingival finish line locations with gingival displacement cords (N = 16 per locations; Fig. 1). All scanning and analysis procedures were performed by an experienced investigator (K.S.).

Sixteen test virtual models acquired per finishing line locations and a reference virtual model were extracted as STL files for interim crown fabrication. In a dental CAD software program (3Shape, Copenhagen, Denmark), the design of interim crowns was performed under the same conditions of a 60-µm cement space. The STL file of the interim crown designed based on the reference virtual model was designated as a CAD reference model (CRM) for the evaluation of intaglio surface trueness (Fig. 1). Interim crowns designed based on the test virtual model were fabricated using a stereolithography 3D printer (ZENITH; Dentis, Daegu, Republic of Korea) with 0° parallel to the vat bottom. In consideration of the printing and repetition accuracy according to the position of the printed object in the vat, the interim crowns produced in four groups were divided into quarters and adjusted to the same position and number when printing once. For the photopolymerization resin for the interim crown, 3D printing resin (For interim crown; Dentis, Daegu, Republic of Korea) was used. For interim crowns after printing, all residual resin was removed according to the manufacturer’s recommendations, and postphotopolymerization was performed using a light-curing unit (CUREDEN; Kwang Myung DAICOM, Seoul, Republic of Korea). All evaluations were completed within 3 h after printing in consideration of the dimensional change according to the time change after printing. The intaglio surface of interim crowns after all posttreatments were scanned using an intraoral scanner (i500; MEDIT, Seoul, Republic of Korea), and the STL file was designated as the CAD test model (CTM) for the evaluation of the intaglio surface trueness (Fig. 1).

Through the evaluation of the intaglio surface trueness, the accuracy of the intaglio surface of interim crowns manufactured according to the finishing line locations was compared (Fig. 1). CRM and CTM alignment and 3D comparison were performed using a 3D inspection software program (Geomagic Control X; 3D Systems, Rock Hill, SC, USA) (Fig. 1). The area of the intaglio surface was segmented based on the margin of CRM. To evaluate the intaglio surface area in detail, it was divided into the marginal, axial, and occlusal regions. CRM and CTM were aligned based on the segmented intaglio surface, and the root mean square was calculated as follows based on all cloud points of the CRM intaglio surface (Eq. 1):

1 $$RMS=\frac{1}{\sqrt{n}}\cdot \sqrt{{\sum }_{i\,\,=\,\,1}^{n}{{D}_{i}}^{2}}$$

where ${D}_{i}$ represents the gap distance of point $i$ of CRM and CTM and *n* is the number of all points evaluated.

### Evaluation of the marginal and internal fit

**Figure 2:**
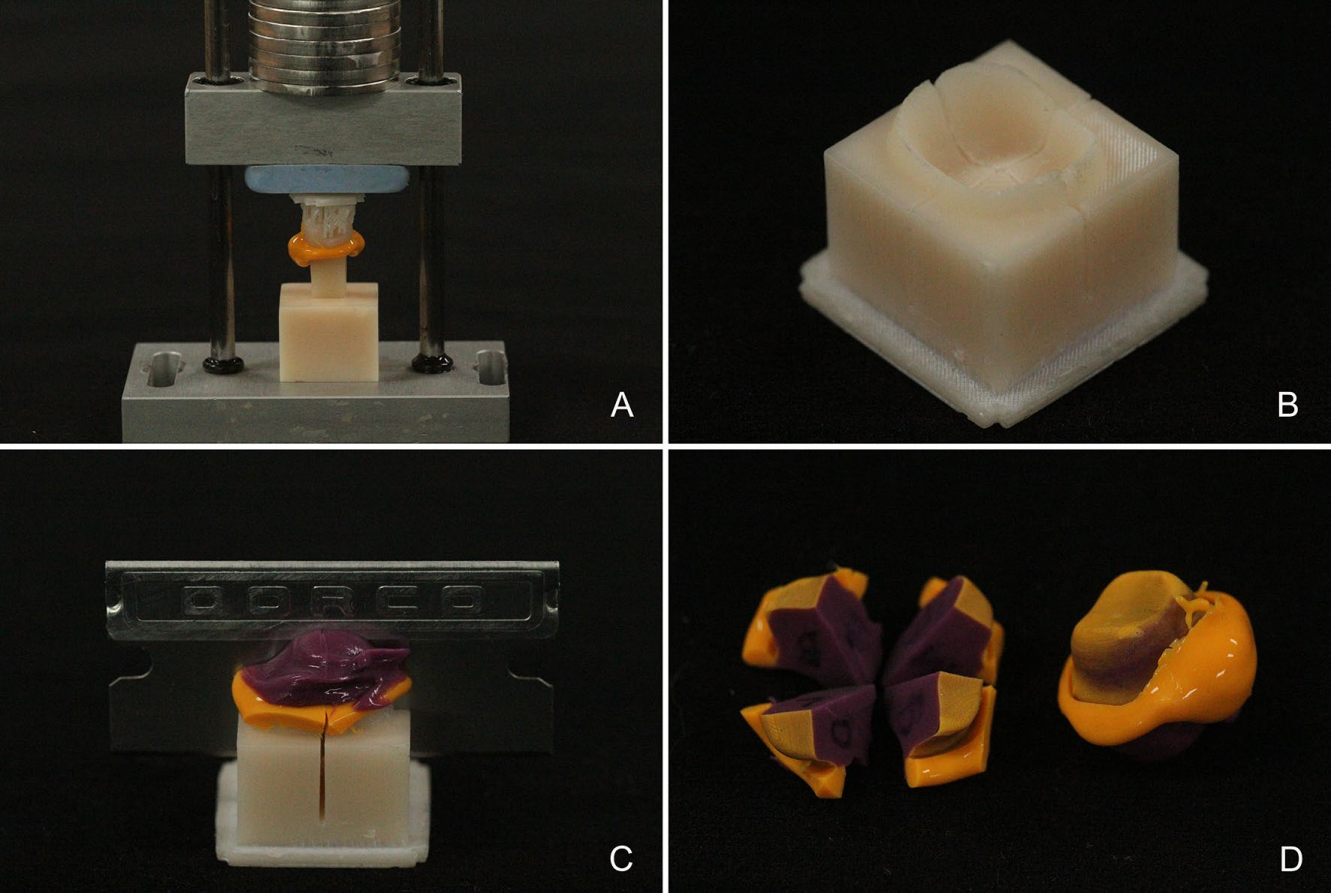
Procedure for the marginal and internal fit using the silicone replica technique. (**a**) Applying constant load for the interim crown with silicone, (**b**) guide template for cutting of the silicone replica, (**c**) cutting of the silicone replica, (**d**) silicone replica.

**Figure 3:**
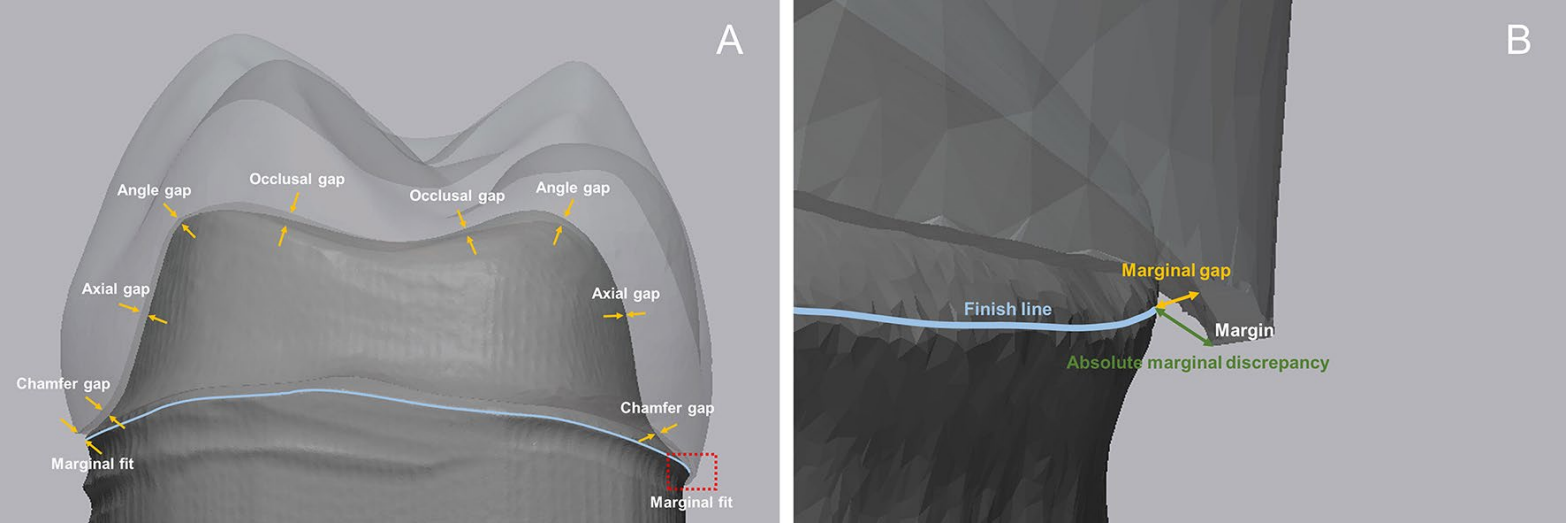
Schematic of the measurement regions of the marginal and internal fit. (**a**) Marginal and internal fit, (**b**) marginal fit.

The silicone replica technique was performed to evaluate the marginal and internal fit of interim crowns. After filling the silicone indicator (Aquasil Ultra XLV; Dentsply Detrey GmbH, Konstanz, Germany) in the intaglio surface of the interim crown and accurately positioning it on the tooth preparation, use a jig capable of continuously applying the force (300 gf) on the occlusal surface. Continuous force was applied until the polymerization of the silicone was completed (Fig. 2a). The silicone indicator attached to the intaglio surface of the interim crown was filled with silicone of a different color, and the silicone replica was separated from the interim crown after polymerization.For identical cutting of the silicone replica, an industrial CAD software program (SolidWorks 2014 software; Dassault Systems SolidWorks Corp., Waltham, MA, USA) was used to design a jig based on CRM, and the jig was fabricated using a 3D printer (Megprinter; Megagen, Daegu, Republic of Korea) (Fig. 2b). The jig for cutting the silicone replica is designed to cut the buccolingual and mesiodistal planes based on the center of the interim crown (Fig. 2c). The distance in the silicone replica (Fig. 2d) was measured using an optical microscope (IMS 1080P; SOMETECH, Seoul, Republic of Korea). As for the measurement point of the marginal fit, the marginal gap (MG), which measures the marginal opening, and absolute marginal discrepancy (AMD), which measures the distance between the finishing line and the margin of the prosthesis, were evaluated (Fig. 3). The measurement points of the internal fit are the chamfer gap, which measures the distance between the center of the chamfer curvature of tooth preparation, angle gap, which measures the distance between the center of the angle curvature, and axial gap, which measures the distance between the center of the chamfer and the angle (Fig. 3). The occlusal gap was evaluated by measuring the distance between the center of the occlusal and middle point of the angle (Fig. 3).

### Statistical analysis

Statistical analysis was performed using a statistical software program (SPSS Ver 25.0; IBM, Chicago, USA) (α = 0.05). Since all the acquired data had a normal distribution, a parametric statistical analysis was used. Statistical comparison of the marginal and internal fit and intaglio surface trueness per groups was performed using one-way analysis of variance and the Tukey HSD test. The correlation between marginal region trueness and marginal fit (AMD and MG) was evaluated using Pearson correlation analysis.

## Results

**Figure 4:**
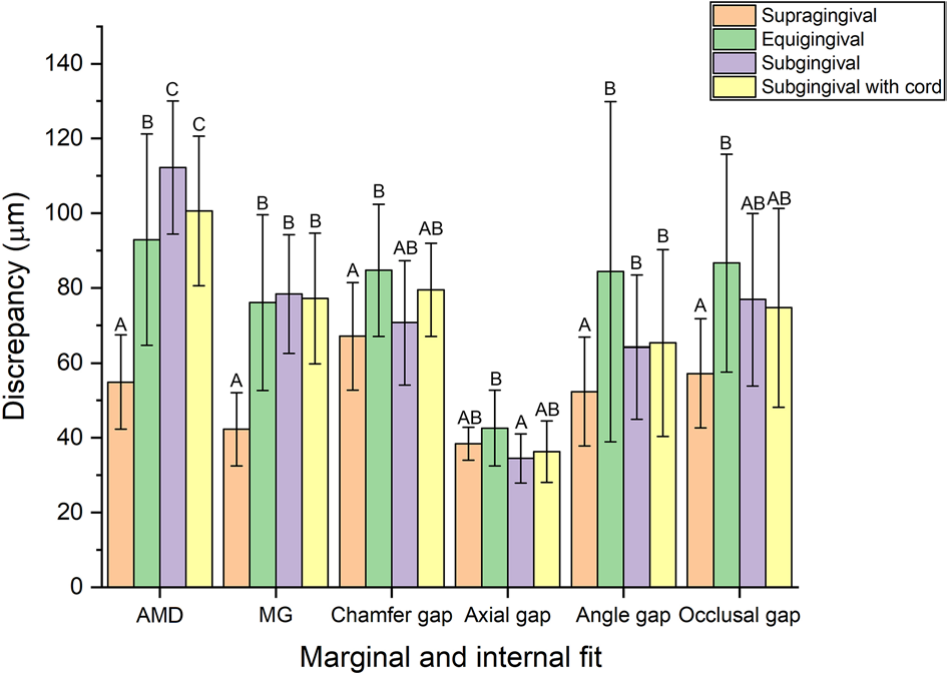
Comparison of the marginal and internal fit of the interim crowns fabricated from tooth preparation scanned at four finish lines. Different letters indicate significant differences among finish line locations by Tukey HSD test, *P* < 0.05. *AMD* absolute marginal discrepancy, *MG* marginal gap.

Significant differences were found in the marginal and internal fit according to finish line locations (*P* < 0.05; Table 1; Fig. 4). Marginal fit showed the lowest value at the supragingival finish line (AMD: 59.4 ± 12.6 µm, MG: 42.3 ± 9.8 µm) (*P* < 0.05; Table 1; Fig. 4) and relatively high values at the subgingival finish line (AMD: 112.2 ± 17.8 µm, MG: 78.4 ± 15.8 µm), but no significant difference was found at other finish line locations (*P* > 0.05; Table 1; Fig. 4). The internal fit showed the lowest value at the supragingival finish line, excluding the axial gap (*P* < 0.05; Table 1; Fig. 4).

**Table 1:** Comparison of marginal and internal fit according to finish line locations.

Marginal and internal fit	Finish line	Mean	SD	95% Confidential interval		Minimum	Maximum	F	P
				Lower	Upper				
AMD	Supra	54.9^A^	12.6	48.3	61.7	32.9	79.1	23.411	< 0.001*
	Equi	92.9^B^	28.3	77.8	108.0	33.5	140.8		
	Sub	112.2^C^	17.8	102.6	121.7	74.6	141.9		
	With cord	100.6^BC^	20.0	90.0	111.4	68.1	130.4		
MG	Supra	42.3^A^	9.8	37.1	47.5	24.4	62.9	16.344	< 0.001*
	Equi	76.1^B^	23.5	63.6	88.6	31.9	122.0		
	Sub	78.4^B^	15.8	70.0	86.9	55.9	112.4		
	With cord	77.2^B^	17.4	67.9	86.5	54.0	106.2		
Chamfer gap	Supra	67.1^A^	14.3	59.5	74.7	33.7	84.4	4.364	0.008*
	Equi	84.7^B^	17.7	75.3	94.1	47.3	117.4		
	Sub	70.7^AB^	16.6	61.9	79.6	42.0	99.6		
	With cord	79.5^AB^	12.5	72.9	86.2	59.4	99.2		
Axial gap	Supra	38.4^AB^	4.4	36.1	40.8	29.2	45.9	3.317	0.026*
	Equi	42.6^B^	10.2	37.2	48.1	26.7	65.5		
	Sub	34.5^A^	6.6	31.0	38.1	22.5	47.8		
	With cord	36.3^AB^	8.2	32.0	40.7	24.4	53.8		
Angle gap	Supra	52.3^A^	14.5	44.6	60.1	33.6	78.6	3.441	0.022*
	Equi	84.4^B^	45.5	60.2	108.6	46.8	232.3		
	Sub	64.2^AB^	19.3	54.0	74.5	41.2	100.9		
	With cord	65.3^AB^	24.9	52.0	78.6	28.7	120.0		
Occlusal gap	Supra	57.2^A^	14.6	49.5	65.0	37.0	87.5	4.2	0.009*
	Equi	86.7^B^	29.1	71.2	102.2	53.1	147.6		
	Sub	76.9^AB^	23.1	64.7	89.3	48.4	126.3		
	With cord	74.7^AB^	26.6	60.6	88.9	47.2	137.4		

*AMD* absolute marginal discrepancy, *MG* marginal gap.

*Significance determined by one-way ANOVA, *P* < 0.05. Different letters indicate significant differences among finish line locations by Tukey HSD test, *P* < 0.05.

**Figure 5:**
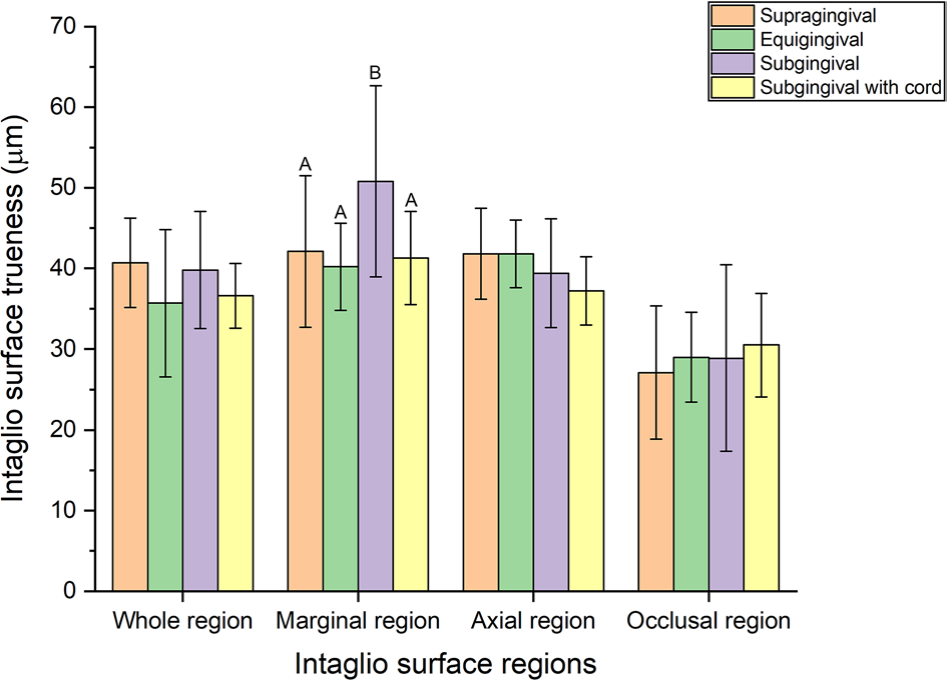
Comparison of the intaglio surface trueness of the interim crowns fabricated from tooth preparation scanned at four finish lines. Different letters indicate significant differences among finish line locations by Tukey HSD test, *P* < 0.05.

Intaglio surface trueness was significantly different in the marginal region (*P* = 0.003), and no significant difference was found in the whole, axial, and occlusal regions (*P* > 0.05; Table 2; Fig. 5). The trueness of the marginal region was highest in the subgingival finish line (50.8 ± 11.9 µm) (*P* < 0.05), but no significant difference was found at other finish line locations (*P* > 0.05; Table 2; Fig. 5).

**Table 2:** Comparison of intaglio surface trueness according to finish line locations.

Region	Finish line	Mean	SD	95% Confidential interval		Minimum	Maximum	F	P
				Lower	Upper				
Whole region	Supra	40.7	5.5	37.8	43.7	31.7	53.2	2.064	0.114
	Equi	35.7	9.1	30.9	40.6	3.5	45.5		
	Sub	39.8	7.3	36.0	43.8	29.2	59.9		
	With cord	36.6	4.0	34.5	38.8	29.6	43.6		
Marginal region	Supra	42.1^A^	9.4	37.1	47.1	30.1	62.3	5.227	0.003*
	Equi	40.2^A^	5.4	37.3	43.1	34.0	51.5		
	Sub	50.8^B^	11.9	44.6	57.2	38.3	82.2		
	With cord	41.3^A^	5.8	38.3	44.5	32.1	52.0		
Axial region	Supra	41.8	5.6	38.8	44.8	35.0	53.4	2.755	0.05
	Equi	41.8	4.2	39.6	44.1	33.4	47.0		
	Sub	39.4	6.8	35.8	43.0	27.7	51.3		
	With cord	37.2	4.2	35.0	39.5	30.4	43.9		
Occlusal region	Supra	27.1	8.2	22.7	31.5	18.1	49.1	0.462	0.71
	Equi	29.0	5.5	26.1	32.0	20.2	38.9		
	Sub	28.9	11.5	22.8	35.1	16.2	61.3		
	With cord	30.5	6.4	27.1	34.0	20.6	41.1		

*Significance determined by one-way ANOVA, *P* < 0.05. Different letters indicate significant differences among finish line locations by Tukey HSD test, *P* < 0.05.

A significant positive correlation was noted between the trueness of the marginal region and marginal fit (AMD and MG) (*P* < 0.05; Table 3).

**Table 3:** Results of the correlation analysis between marginal fit and trueness of the marginal region.

Trueness		Marginal fit	
		AMD	MG
Marginal region	P	0.004*	0.046*
	CC	0.351	0.25

*AMD* absolute marginal discrepancy, *MG* marginal gap, *CC* correlation coefficient.

*Significance determined by Pearson correlation analysis, *P* < 0.05.

## Discussion

In this study, interim crowns were fabricated from tooth preparations in four finish line locations (supragingival, equigingival, subgingival, and subgingival with a cord finish line), and marginal and internal fit and intaglio surface trueness were evaluated. Significant differences in the marginal and internal fit of interim crowns fabricated at four finish line locations were observed, so the null hypothesis was rejected (*P* < 0.05; Table 1). However, the intaglio surface trueness had a significant difference only in the marginal region, so the null hypothesis was partially rejected (*P* = 0.003; Table 2). Therefore, these results imply that finish line locations during intraoral scans may affect the marginal and internal fit of interim crowns and the intaglio surface trueness of the marginal region.

In many studies, the marginal fit of dental prosthesis fabricated using an intraoral scanner was evaluated compared with conventional methods for application to dental clinic. Su et al.^11^ evaluated 3-unit zirconia fixed dental prostheses fabricated using an intraoral scanner (TRIOS2) and reported a better marginal fit (AMD) in the digital group (64 ± 16 µm) than in the conventional group (76 ± 18 µm). Arezoobakhsh et al.^12^ evaluated 3-unit zirconia frameworks fabricated using intraoral scanners (TRIOS3 and CS3600) and reported better marginal fit (MG) in the digital group (TRIOS3, 60 ± 15 µm; CS3600, 55 ± 13 µm) than in the conventional group (91 ± 40 µm). The equigingival finish line location was applied in study of Su et al.^11^ and the supragingival finish line location in study of Arezoobakhsh et al.^12^. The comparison is difficult owing to differences in manufacturing materials and methods used in this study, but all digital groups showed a marginal fit within a mean 120 µm. In this study, the marginal fit of the subgingival finish line (AMD, 112.2 ± 15 µm) was clinically acceptable, but at 95% confidential intervals, the case exceeded 120 µm (Table 1). Of course, a marginal fit exceeding 120 µm in the fabrication of an interim crown that is not a permanent dental prosthesis is not impossible to be applied clinically, but more clinical attention is required in the subgingival finish line.

In previous studies, intaglio surface trueness was evaluated for various purposes^17–19^. Wang et al.^20^ evaluated the intaglio surface trueness of zirconia crowns fabricated with 3D printing and verified the volumetric stability of the fabricated zirconia crowns. In a previous clinical study, virtual models of crowns before and after intraoral adjustment were superimposed to assess intaglio surface trueness, and the intraoral adjustment of crowns was verified^21^. In this study, to evaluate the effect of the four finish line locations, crowns fabricated in four finish line locations and crowns designed in a reference tooth preparation were superimposed and the intaglio surface trueness was evaluated (Fig. 1). Only in the marginal region, the intaglio surface trueness was significantly different according to the finish line locations and significantly higher trueness in the subgingival finish line. This means that in the subgingival finish line, there may be an inaccurate reproduction of the marginal region of the interim crowns. Accordingly, the correlation between the trueness of the marginal region and the marginal fit (AMD and MG) was analyzed and a significant positive correlation was found (*P* < 0.05; Table 3). In light of these results, the result of an inaccurate marginal fit could be seen in the subgingival finish line, since there may be an inaccurate marginal region of interim crowns. Therefore, fabrication of an interim crown by intraoral scan is not recommended for the subgingival finish line.

Nedelcu et al.^33^ evaluated the effect on the quality of the scanned finish line using intraoral scanners according to the finish line locations and confirmed that it was difficult to clearly distinguish the gingiva from the finish line in the subgingival finish line. In another study, the effect of finish line locations on scan accuracy was evaluated, and the supragingival finish line or a use of gingival displacement cord was recommended for clinically acceptable scan accuracy (< 100 µm)^34^. However, in this study, the use of the gingival displacement cord at the subgingival finish line did not affect the marginal and internal fit results (*P* > 0.05; Table 1; Fig. 4). For this reason, previous studies have reported that the accuracy of the supragingival finish line was improved using a gingival displacement cord; however, except for the supragingival finish line (accuracy, 33.6 ± 1.8 µm), the scan accuracy was still inaccurate in the equigingival (accuracy, 127.6 ± 14.7 µm) and subgingival with cord (accuracy, 68.5 ± 7.3 µm)^34^. In light of these results, consensus is still needed on the effect of scan accuracy on marginal and internal fit through additional studies.

Previous studies have reported the accuracy of intraoral scanners and 3D printers^17–25^. The intraoral scanner (i500; MEDIT) used in the present study for scanning of preparations and intaglio surfaces reported an accuracy of 20–30 µm with respect to a single tooth according to previous study^35^. In the present study, taking these errors into account, all scans were performed by one experienced investigator (K.S.), and the intraoral scanner was calibrated every time before the experiment. In addition, a 3D printer with SLA technology was used for the fabrication of interim crowns in the present study, and a previous study reported excellent accuracy in 3D printer with SLA technology^36^. However, to verify the accuracy in the present study, CAD data was designated as a reference and the fabrication precision was further evaluated by comparing it with the scan data of intaglio surfaces. As a result, the mean fabrication precision of 28.1 ± 4.9 µm was shown, and there was no significant difference in fabrication precision among finish line groups (F = 1.179; *P* = 0.325). However, errors of intraoral scanners and 3D printers may be reflected in the results, and additional studies should be conducted.

This study has several limitations. Although the clinical environment was reproduced in an in vitro environment, there are still differences in the actual teeth and gingiva. Therefore, additional clinical trials should be conducted to verify the effect of finish line locations. Moreover, because the results for various intraoral scanners are insufficient, additional studies have to be conducted to derive more complex results.

## Conclusion

Finish line locations influenced the marginal and internal fit of interim crowns. The marginal fit showed the best results in the supragingival finish line, but AMD showed the worst fit in the subgingival finish line. In addition, the finish line locations affected the trueness of the marginal region and showed the worst fabrication reproducibility of the marginal region in the subgingival finish line. This is because the trueness of the marginal region had a positive correlation with the marginal fit, and interim crowns fabricated on the subgingival finish line resulted in inaccurate marginal fit due to poor fabrication reproducibility of the marginal region. Therefore, the use of an intraoral scanner should be decided on the clinical situation and needs.

## Data Availability

All outcome data are available as summary measures or representative images in the main text or the extended data. The raw datasets generated analyzed during the current study are available from the corresponding author on reasonable request.
